# Differential pulse voltammetry detection of Pb(ii) using nitrogen-doped activated nanoporous carbon from almond shells

**DOI:** 10.1039/c9ra03925b

**Published:** 2019-08-01

**Authors:** Yiliyasi Baikeli, Xamxikamar Mamat, Nuerbiya Yalikun, Ying Wang, Mengfei Qiao, Yongtao Li, Guangzhi Hu

**Affiliations:** Key Laboratory of Chemistry of Plant Resources in Arid Regions, State Key Laboratory Basis of Xinjiang Indigenous Medicinal Plants Resource Utilization, Xinjiang Technical Institute of Physics and Chemistry, Chinese Academy of Science Urumqi 830011 China xamxikmr@ms.xjb.ac.cn yongtao@ms.xjb.ac.cn guangzhihu@ms.xjb.ac.cn; University of Chinese Academy of Sciences Beijing 100049 China

## Abstract

Almond shell-based charcoal was prepared by carbonizing almond shells in a nitrogen atmosphere. Nanoporous carbon (NPC) was formed *via* activating the obtained charcoal using potassium hydroxide as an activating agent, followed by the synthesis of nitrogen-doped nanoporous carbon (N-NPC) *via* a hydrothermal reaction using urea as the nitrogen source. The obtained N-NPC possessed a large surface area (1075 m^2^ g^−1^), narrow pore-size distribution (1–2 nm) and nitrogen content reaching 2.23 wt%. Using N-NPC with Nafion to modify a glassy carbon electrode, a highly sensitive electrochemical sensor was fabricated for the determination of Pb(ii) in aqueous solutions with differential pulse anodic stripping voltammetry (DPASV). The peak current of Pb(ii) showed linearity over concentrations from 2.0 to 120 μg L^−1^ and the detection limit (S/N = 3) was estimated to be 0.7 μg L^−1^ for Pb(ii), which was 15-fold lower than the guideline value of drinking water given by the World Health Organization (WHO). The experimental data indicated that this easy and low-cost method is an accurate and fast method for the detection of trace Pb(ii).

## Abstract 2

Almond shell-based charcoal was prepared by carbonizing almond shells in a nitrogen atmosphere.

## Graphical abstract

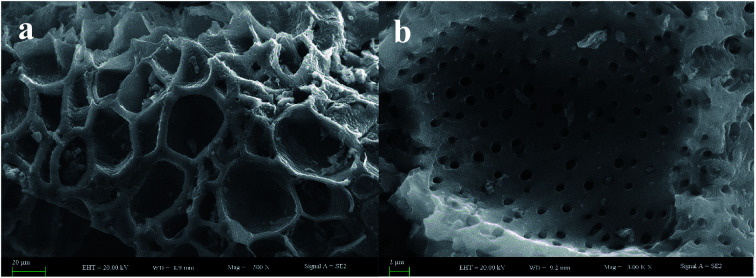

## Introduction

Heavy metals such as cobalt, copper, iron, manganese, and zinc have an important role in the bodies of living organisms and are essential in trace amounts for their metabolism and maintenance.^1,2^ However, excessive levels or even small doses of very toxic metals such as lead (Pb), cadmium (Cd), mercury (Hg), arsenic (As), and antimony (Sb) can cause serious problems in the environment and their accumulation in the human body can cause diseases of the central nervous system, liver and kidneys, or skin, bones, and teeth.^3,4^ Thus, detecting trace metals is necessary and urgent for the health and safety of humans.^5^ So far, several techniques and methods have been utilized to detect heavy metal ions, including atomic absorption spectroscopy, atomic fluorescence spectrometry, hyper-Rayleigh scattering, and inductively coupled plasma mass spectrometry.^6,7^ Although reliable results with high sensitivities can be obtained by these spectroscopic methods, disadvantages such as the high costs of these instruments and complex operation procedures may limit their applications.^8,9^ Electrochemical techniques have been considered as efficient alternative techniques due to their advantages of low cost, high sensitivity and good portability.^10^ For the detection of trace heavy metal ions in the environment, anodic stripping voltammetry (ASV) is one of the most effective options as a sensitive, low-cost, and fast method. The sensitivity of ASV can be effectively improved by the chemical modification of the electrode surface.^11,12^ According to some previous references, different carbon-based nanomaterials such as graphene,^13,14^ carbon nanotubes^15,16^ and porous carbon^17^ have been used to fabricate modified electrodes because of the high surface area, low charge-transfer resistance, and excellent chemical stability.^18,19^ However, the method of the preparation of such types of carbon materials requires expensive raw materials, considerable time and energy, and complicated procedures. In contrast, biomass wastes are very cheap and easily available, which make them potential raw materials for the preparation of porous carbon materials having good electrochemical performances.^20,21^ Almonds are one of the important nut fruits grown in the south of China (Xinjiang) and they are widely consumed. Almond nutshells are abundant waste materials and easy to collect for reuse. In this study, almond nutshells were used to prepare N-doped nanoporous carbon (N-NPC) for its application as an electrode modifier.

As we know, active carbons are widely used in gas adsorption and air/water cleaning, but there are no reports on N-doped nanoporous carbons derived from almond shells used in the electrochemical detection of trace metals. Here, by selecting differential pulse anodic stripping voltammetry (DPASV) with high sensitivity and the best selectivity, for the first time, we reported that nitrogen-doped nanoporous carbons (N-NPC) prepared from almond shells could be successfully applied for the detection of Pb(ii) ions in aqueous solutions. In comparison with some previously reported materials, the nitrogen-doped nanoporous carbon fabricated from almond shells was found to be one of the most promising candidates for the rapid determination of Pb(ii) in water samples.

## Experimental

### Chemical reagents and instruments

We purchased almonds from the local market and obtained the shells. By mixing the stock solutions of 0.1 M NaAc and HAc, 0.1 M acetate buffer solution (HAc–NaAc) was prepared. Standard Pb(ii) solution (1000 mg L^−1^) was received from NCS Testing Technology Co. Ltd. (Beijing, China), and 5% Nafion stock solution was bought from Sigma-Aldrich (Shanghai, China). All chemical reagents received were of analytical grade. For all electrochemical tests, solutions were prepared in ultrapure water (≥18.2 MΩ cm).

A RST 5000C workstation (Zhengzhou Shiruisi Technology Co., Ltd. Zhengzhou, China) was used for electrochemical analysis. For carbonization and activation, we used a pipe furnace (GSL-1500X, Hefei Kejing Material Co., Ltd. Hefei, China). For the characterization of N-NPC samples, various techniques such as X-ray diffraction (X-ray D/max-2200vpc, Rigaku Corporation, Japan), Raman spectroscopy (confocal microprobe Raman system, HR800, Jobin Yvon, France), X-ray photoelectron spectroscopy (XPS, Thermo Scientific Escalab 250Xi, China), nitrogen sorption (NOVA 2000e, USA) and scanning electron microscopy (SEM, FEI Verios 460 L XHR Germany) were used.

### Preparation of nanoporous carbon (NPC)

Almond shells were washed with water and dried in an oven at 100 °C for 12 h to remove the moisture content. The dried shells were ground by a pestle and mortar and carbonized and activated in a pipe furnace. First, 8 g of dried almond shells was placed in the furnace tube and nitrogen gas was passed for about 20 minutes. The furnace temperature was increased at a rate of 5 °C min^−1^ from room temperature to 450 °C and held at this temperature for 5 h. After pyrolysis, the furnace was cooled down to room temperature and 3.34 g of charcoal was obtained. The above process from start to finish was carried under nitrogen gas flushing. After cooling down, we obtained the carbonized sample (1 g) and soaked it in KOH solution (mass ratio 1 : 1) for about 24 h by stirring, followed by drying at 80 °C in the oven for 10 h. Then, we recarbonized the resulting samples in a pipe furnace in a nitrogen atmosphere. During recarbonization, the furnace was heated at a rate of 5 °C min^−1^ from room temperature to 600 °C and held at this temperature for 2 h. After cooling down to room temperature under the nitrogen flow, the resulting material was washed with an HCl solution and deionized water several times. Then, the pyrolyzed material was dried at 80 °C in the oven for 10 h and the final product of 0.726 g nanoporous carbon (NPC) was obtained.

### Preparation of N-NPC

To prepare N-doped nanoporous carbon (N-NPC), 250 mg urea as the nitrogen source was dissolved in 20 ml of anhydrous ethanol/deionized water mixed solution (volume ratio 1 : 1) and 50 mg as-prepared NPC was added in the solution, followed by sonication for 30 min. The resulting solution was kept in a stainless steel Teflon reactor and reacted for 12 h in the oven at 180 °C.

### Preparation of N-NPC-Nafion/GCE

To modify the electrode, initially, a glassy carbon electrode (GCE, 3 mm diameter, 0.07 cm^2^ surface area) was attentively polished on a polishing cloth (0.3 and 0.05 μm alumina slurry). Then, the polished electrode was successively sonicated for 3 min with ultrapure water and ethanol and was dried under nitrogen gas. Next, 1.5 mg N-NPC dispersed in 1 ml of deionized water/isopropyl alcohol/5 wt% Nafion (volume ratio 7.6 : 1.9 : 0.5) solution was sonicated for 30 min to get a homogenous suspension, and 5 μl suspension was casted on the polished GCE surface. The final N-NPC-Nafion/GCE was obtained after drying for 12 h at room temperature.

### Electrochemical measurements

All electrochemical analyses were performed using the RST 5000C workstation. Three-electrode working systems with N-NPC-modified GCE as the working electrode, Ag/AgCl electrode (in saturated KCl solution, Gaoss Union, China) as the reference electrode and platinum wire as the counter electrode were chosen. For the determination of Pb(ii), N-NPC/Nafion/GCE obtained by the DPASV method in 0.1 M acetate buffer (HAc–NaAc) was used. Initially, Pb(ii) ions were electrochemically deposited on the surface of N-NPC/Nafion/GCE at −1.3 V for 330 s under magnetic stirring. After that, the electrolyte was held quiet for 10 s; then, differential pulse voltammetry (DPV) in the potential range from −1.0 V to −0.4 V with amplitude of 50 mV, pulse width of 50 ms and potential step of 4 mV was conducted.

## Results and discussion

### Characterization of N-NPC

**Fig. 1:**
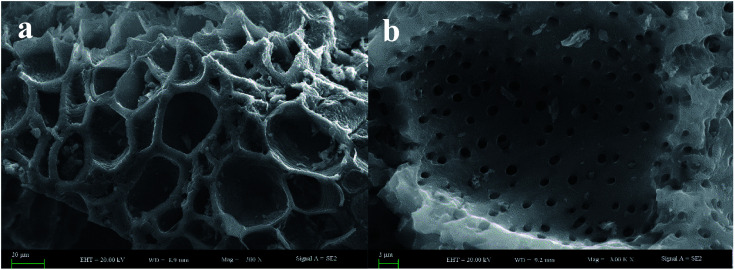
(a and b) SEM images of N-NPC.

**Fig. 2:**
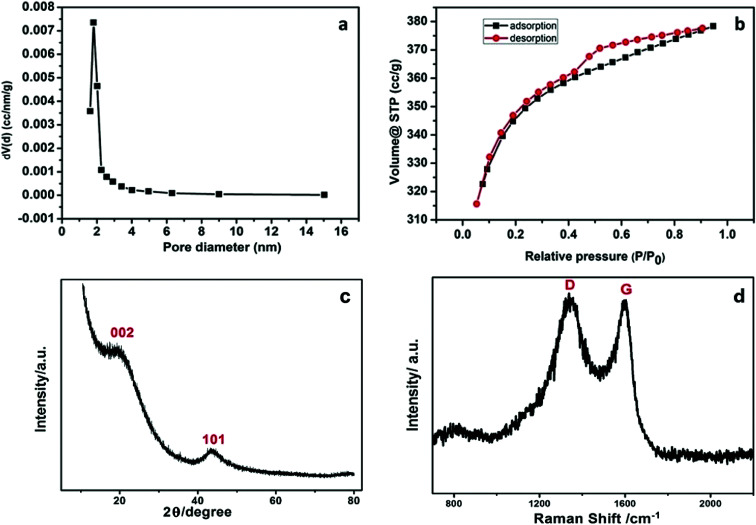
(a) Pore-size distribution, (b) N_2_ sorption isotherm, (c) XRD spectrum and (d) Raman spectrum of N-NPC.

**Fig. 3:**
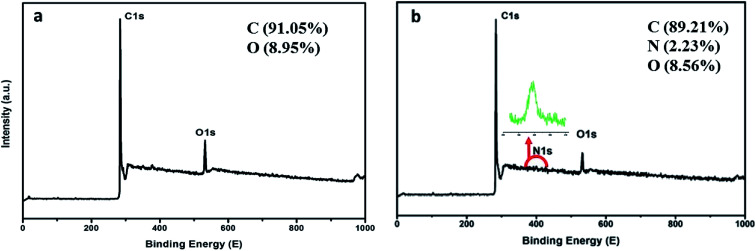
XPS spectra of (a) NPC and (b) N-NPC.

The morphological and elementary properties of N-NPC were characterized by SEM. The specific surface area and pore structure of the samples were characterized by nitrogen adsorption and desorption experiments at 77 K. The specific surface area was calculated from the nitrogen adsorption isotherm using the Brunauer–Emmett–Teller (BET) method and the pore size distribution was measured by Barrett–Joyner–Halenda theories. As illustrated in the SEM (Fig. 1a and b) images, there are abundant micropores (shown in Fig. 2a, the main pore diameters are 1–2 nm) in the honeycomb-like surface of N-NPC. These abundant micropores have been found to be effective in increasing specific surface area^22^ and providing several interpenetrating channels to infiltrate the electrolyte into the electrode materials and shorten the distance of Pb(ii) transport.^23^ In order to understand the graphitization degree, wide-angle X-ray diffraction (XRD) was carried out. As shown in Fig. 2c, two broad and small peaks at 20.2° and 43.5° correspond to the (002) and (101) planes, which are generally observed for carbon materials. The weak (002) peak demonstrated the low degree of graphitization and low concentration of parallel single layers on N-NPC.^24^ To obtain further information about the structure of N-NPC, Raman spectroscopy was carried out. As shown in Fig. 2d, there are two distinct peaks at 1339 and 1596 cm^−1^. As we know, the D band (1339 cm^−1^) and G band (1596 cm^−1^) are related to the structural defects caused by sp^3^-hybridized carbons and crystalline graphite, respectively.^25^ It is generally considered that the relative intensity ratio of D and G bands (*I*_D_/*I*_G_) corresponds to the amount of defect sites on carbon materials.^26^ The *I*_D_/*I*_G_ value for N-NPC was calculated to be 2.99. This result indicated that there were large amounts of defective sites in the structure of the as-prepared N-NPC.^27^ The coexistence of elements in NPC and N-NPC was confirmed by XPS. Fig. 3a demonstrates the XPS spectrum of NPC, which confirmed the coexistence of C (91.05%) and O (8.95%); as shown in Fig. 3b, three elements are present in N-NPC: C (89.21%), O (8.56%), and N (2.23%). The C1s (283.8 eV), O1s (531.9 eV) and N1s (399.4 eV) spectra were also determined.

**Fig. 4:**
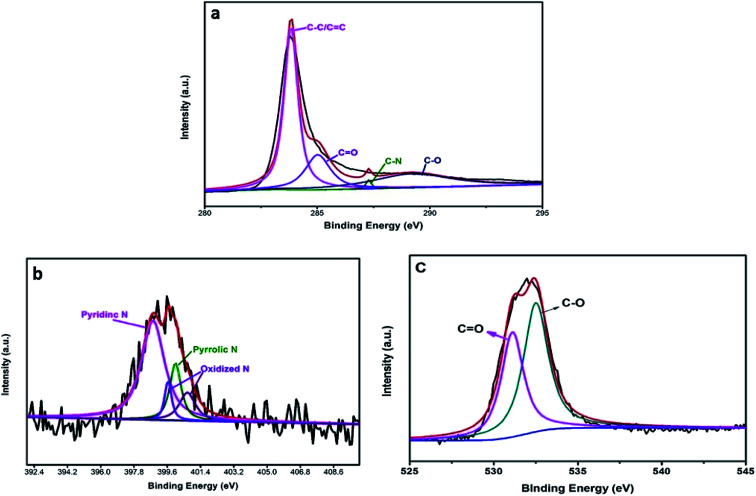
High-resolution spectra of (a) C1s, (b) N1s and (c) O1s.

Fig. 4a shows that the high-resolution C1s peak corresponds to four curves at 284.6 eV, 286.0 eV, 286.4 eV, and 288.8 eV, which are related to C–C/C=C, C–N, C–O, and C=O bonds, respectively; this is in accordance with the results of a previous report.^28^ Additionally, Fig. 4b shows the high-resolution N1s peak, which corresponds to different peaks that are characteristics of N such as pyridinic N (398.3 eV) and pyrrolic N (399.4 eV); the other two peaks (404.6 eV, 406.3 eV) are ascribed to oxidative species.^29^ The O1s peak (Fig. 4c) was decomposed into different peaks to characterize the states of O1s. The results confirmed that the major C–O bonding configuration was C=O (530.7 eV) as well as C–O (533.9 eV).^30^

### Electrochemical performance of different electrodes

**Fig. 5:**
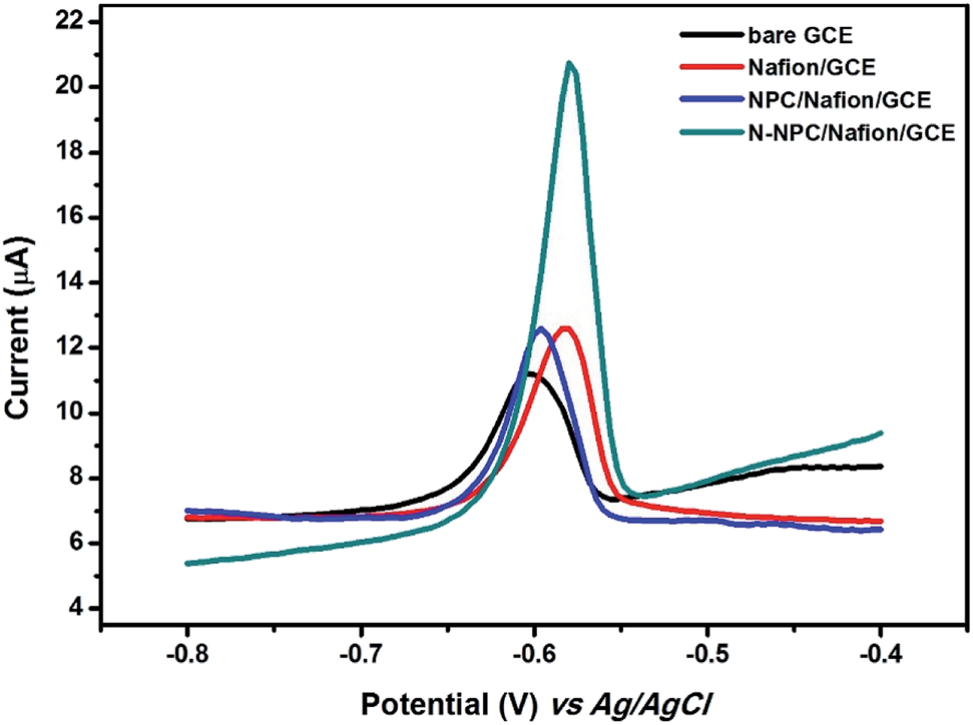
DPASV curves at different electrodes in 0.1 M HAC–NaAc buffer (pH = 5.0) containing 100 μg L^−1^ of Pb(ii).

The electrochemical performances of different electrodes such as bare GCE, Nafion/GCE, and N-NPC-Nafion/GCE were determined by recording differential pulse anodic stripping voltammetry (DPASV) measurements in order to find out the electrochemical sensitivity of N-NPC for Pb(ii) detection. As shown in Fig. 5, the stripping peak current of bare GCE is 3.965 μA for Pb(ii). After casting the Nafion polymer, the peak current of Pb(ii) on Nafion/GCE increased to 5.86 μA; this was because of the polyelectrolyte polymer having negative anion groups interconnected with heavy metals due to the static accumulation of positive ions.^31^ The peak current of NPC/Nafion/GCE was 6.012 μA, which means that NPC did not cause significant changes in the peak current for Pb(ii). The peak current of N-NPC/Nafion/GCE for Pb(ii) reached 13.054 μA. Compared to the other electrodes, N-NPC/Nafion/GCE exhibited the best electrochemical performance because of the following reasons: (i) increase in the electroactive surface area due to the nanoporous structure of N-NPC and (ii) increased electroconductivity of N-NPC due to the doped nitrogen atoms.^22^

### Optimal parameters of measurements

**Fig. 6:**
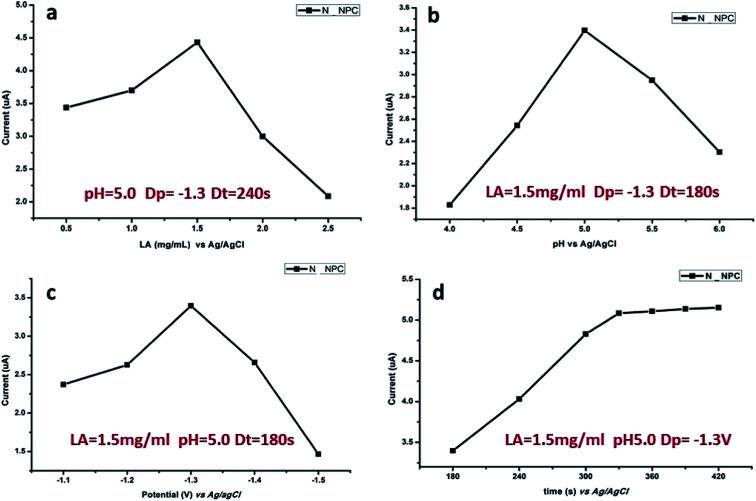
Influence of various experimental parameters on stripping response of 50 μg L^−1^ of Pb(ii): (a) load amount of N-NPC on GCE, (b) pH value, (c) deposition potential and (d) deposition time.

The response signal of DPASV is mostly dependent on experimental conditions such as the pH value of buffer solution, loading concentration of N-NPC, and deposition time and potential. The optimization of the experimental conditions is necessary. In this study, the following conditions were optimized in sequence: loading amount of sample, pH value of the buffer solution, deposition potential and deposition time. As shown in Fig. 6, the optimal load is 7.5 μg (Fig. 6a), the best pH value of buffer is 5.0 (Fig. 6b), and the best deposition potential (Fig. 6c) and time (Fig. 6d) are −1.3 V and 330 s, respectively.

### Analyses for the determination of Pb(ii)

**Fig. 7:**
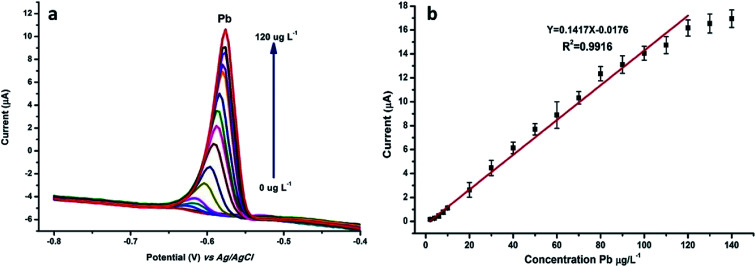
(a) DPASV and (b) calibration plots of stripping peak current were tested in 0.1 M HAc–NaAc buffer solution by changing the concentration of Pb(ii) from 2 to 120 μg L^−1^.

N-NPC/Nafion/GCE was used for the detection of Pb(ii) ions with the DPASV method under optimal conditions. The measurements were obtained using varying concentrations of Pb(ii). Fig. 7a indicates that the potential at about −0.6 V corresponds to the peak current of Pb(ii). As shown in Fig. 7b, the peak current of Pb(ii) linearly increases in a concentration range from 2.0 to 120 μg L^−1^ according to the relevant equation *y* = 0.1417*x* − 0.0176 (*R*^2^ = 0.9916, *y*: current (μA), *x*: concentration (μg L^−1^)); this indicates that there is an excellent and wide linear correlation between the Pb(ii) concentration and peak current. The detection limit (S/N = 3) of Pb(ii) was calculated to be 0.7 μg L^−1^, which was about 15 times below that of the WHO standard for drinking water.

As shown in Table 1, the detection limit of N-NPC/Nafion/GCE is lower than that of the other materials. Although the deposition time is not too short, the linear range is also wider than that of other materials except for GO/DTT/Nafion and G/PANI/PS/SPCE. In view of these aspects, it can be considered that N-NPC/Nafion/GCE is a promising material for the detection of Pb(ii).

**Table 1:** Comparison of the analytical performances of different sensors toward Pb(ii)

Electrode	Method	Linear range	Limit of detection	Ref.
		(μg L^−1^)	(μg L^−1^)	
OMC^a^-IL-chitosan/CILE^b^	DPASV	10–290	5.2	32
Diatomite-MPTMS^c^/GCE	DPASV	20–150	6.9	33
*In situ* Sb^d^SPCE^e^	DPASV	16.8–62.6	5	34
CB-15-crown-5^f^/GEC	DPASV	10.9–186.5	3.3	35
Bi-xerogel^g^/Nafion/GCE	SWASV	1.0–20.7	1.3	36
GO^h^/DTT^i^/Nafion	SWASV	1–2500	1.9	37
G^j^/PANI^k^/PS^l^/SPCE^e^	SWASV	10–500	3.3	38
Nafion-MPCS^m^-CO^n^-Cys^o^-GCE	SWASV	20.7–165.7	56.2	39
RGO^p^/Bi/CPE^q^	DPASV	20–120	0.55	14
N-NPC-Nafion/GCE	DPASV	2–120	0.7	This work

a Ordered mesoporous carbon.

b Carbon ionic-liquid electrode.

c (3-Mercaptopropyl)trimethoxysilane.

d *In situ* antimony.

e Screen-printed carbon electrode.

f 4-Carboxybenzo-15-crown-5.

g Bismuth-xerogel.

h Graphene oxide.

i Diaminoterthiophene.

j Graphene.

k Polyaniline.

l Polystyrene.

m Ordered three-dimensional macroporous carbon spheres.

n Covalently.

o Cysteine.

p Reduced graphene oxide.

q Carbon paste electrode.

### Interference

Anti-interference is a very important parameter in electrochemical detection. Interfering ions coexisting with Pb(ii) can influence the electrochemical sensing abilities of modified electrodes. Thus, it was necessary to explore the selectivity of N-NPC-Nafion/GCE. Ten varying coexisting interfering ions (Ca^2+^, Mg^2+^, Al^3+^, Fe^2+^, Fe^3+^, Zn^2+^, Cu^2+^, Co^2+^, Cr^3+^, Mn^2+^) with concentrations higher than that of Pb(ii) are shown in Table 2. Except for Cu^2+^ ions, most of them demonstrated very weak interferences, which were proven based on these results. The peak current variations of interfering ions were less than 10%, but 1-fold Cu^2+^ led to the decrease in the peak current by 38.7%. This phenomenon may be due to the formation of an inter-metallic Cu compound with Pb in the pre-concentration step. The Cu^2+^ interference could be overcome by adding some ferrocyanide before detection.^40^

**Table 2:** Effect of different interfering ions (different concentrations) towards 50 μg L^−1^ Pb(ii) detection^a^

Salts	Interference	Contribution (%)
		(Pb(ii) = 100%)
CaCl_2_·2H_2_O	Ca^2+^ 100-fold	−5.75%
MgSO_4_·7H_2_O	Mg^2+^ 100-fold	−2.43%
Fe(SO_4_)·7H_2_O	Fe^2+^ 5-fold	−1.85%
FeCl_3_·6H_2_O	Fe^3+^ 5-fold	−6.98%
Al_2_(SO_4_)_3_·18H_2_O	Al^3+^ 100-fold	−2.86%
Zn(NO_3_)_2_·6H_2_O	Zn^2+^ 5-fold	4.71%
Co(NO_3_)_2_·6H_2_O	Co^2+^ 5-fold	9.69%
Cr(NO_3_)_3_·9H_2_O	Cr^3+^ 10-fold	−3.54%
MnSO_4_·H_2_O	Mn^2+^ 100-fold	−4.51%
Cu(NO_3_)_2_·3H_2_O	Cu^2+^ 1-fold	−38.7%

a Tests were done in the best conditions and for each electrode, the test was repeated three times.

### Repeatability and reproducibility of N-NPC-Nafion/GCE

**Fig. 8:**
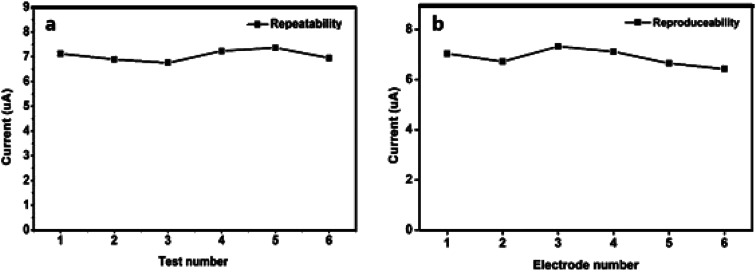
(a) Three electrodes (every single one was tested six times) were used to evaluate the repeatability of N-NPC/Nafion/GCE; (b) six independent electrodes (each one was triplicated) were used to evaluate reproducibility. The experiment parameters were as follows: optimal load: 7.5 μg; optimal pH value: 5.0; optimized deposition potential: −1.3 V (*E vs.* Ag/AgCl); optimal deposition time: 330 s. DPASV was obtained at the scan rate of 0.1 V s^−1^.

The repeatability and reproducibility of N-NPC/Nafion/GCE for Pb(ii) detection were investigated in a buffer solution containing 50 μg L^−1^ Pb(ii) by using the DPASV method. Three new N-NPC/Nafion/GCE samples were prepared and applied to investigate repeatability (each electrode was tested six times), and the relative standard deviation (RSD) value was calculated to be 6.4% for Pb(ii). As shown in Fig. 8a, N-NPC/Nafion/GCE has good measurement stability toward Pb(ii). Additionally, to confirm the reproducibility of N-NPC-Nafion/GCE, six electrodes (each electrode was tested three times) were tested at the same conditions, as shown in Fig. 8b. The RSD value was calculated to be about 7.6%. The lowest RSD value with respect to repeatability and reproducibility makes the electrode a potentially viable candidate to be used for real-time analysis.

### Analysis of real samples

To investigate the practical applicability of N-NPC/Nafion/GCE, tap water was applied. For the tap water, the electrochemical Pb(ii) signal is not measurable. As exhibited in Table 3, after the addition of 7, 37, and 77 μg L^−1^ Pb(ii), the peak current increased sharply. The values of the electrochemical detection recovery were 100.77%, 96.25%, and 102.25%; these completely verified the accuracy and reliability of the modified electrode.

**Table 3:** Detection results of Pb(ii) in tap water with the modified GCE

Sample	Added	Found^a^	Recovery	RSD	ICP-MS
	(μg L^−1^)	(μg L^−1^)	(%)	(%, *n* = 3)	(μg L^−1^)
Polluted tap water	7	7.05 ± 0.5	100.77	7.1	7.23
	37	35.6 ± 3.4	96.25	9.5	39.6
	77	78.7 ± 11.3	102.25	14.4	76.8

a Mean value ± standard deviation value.

## Conclusion

A nanoporous carbon was prepared by directly carbonizing almond shells. Subsequently, nitrogen atoms were doped in the as-obtained porous carbon, followed by the modification of GCE by the N-doped nanoporous carbon, which was applied for Pb(ii) determination by using the DPASV technique. Due to the high content of nitrogen, remarkable nanoporous structure, and large surface area, the N-doped nanoporous carbon-casted GCE demonstrated an excellent electrochemical performance towards Pb(ii), showing good selectivity, a wide linear range, low limit of detection, remarkable reproducibility and good repeatability. The optimal detection conditions of Pb(ii) were investigated and also, its real application was explored. Furthermore, the tap water recoveries indicated that this nanomaterial is a good candidate for monitoring Pb(ii) in running water. However, based on the interference study results, the N-doped nanoporous carbon is not applicable for water conditions containing Cu^2+^ ions.

## Conflicts of interest

The author(s) declare that they have no competing interests.

## Supplementary Material
